# Environment- and eye-centered inhibitory cueing effects are both observed after a methodological confound is eliminated

**DOI:** 10.1038/srep16586

**Published:** 2015-11-13

**Authors:** Tao He, Yun Ding, Zhiguo Wang

**Affiliations:** 1 Center for Cognition and Brain Disorders, Hangzhou Normal University, Hangzhou, 311121, China; 2 Zhejiang Key Laboratory for Research in Assessment of Cognitive Impairments, Hangzhou, 311121, China

## Abstract

Inhibition of return (IOR), typically explored in cueing paradigms, is a performance cost associated with previously attended locations and has been suggested as a crucial attentional mechanism that biases orientation towards novelty. In their seminal IOR paper, Posner and Cohen (1984) showed that IOR is coded in spatiotopic or environment-centered coordinates. Recent studies, however, have consistently reported IOR effects in both spatiotopic and retinotopic (eye-centered) coordinates. One overlooked methodological confound of all previous studies is that the spatial gradient of IOR is not considered when selecting the baseline for estimating IOR effects. This methodological issue makes it difficult to tell if the IOR effects reported in previous studies were coded in retinotopic or spatiotopic coordinates, or in both. The present study addresses this issue with the incorporation of no-cue trials to a modified cueing paradigm in which the cue and target are always intervened by a gaze-shift. The results revealed that a) IOR is indeed coded in both spatiotopic and retinotopic coordinates, and b) the methodology of previous work may have underestimated spatiotopic and retinotopic IOR effects.

Inhibition of return (IOR), first demonstrated by Posner and Cohen^1^ with a spatial cueing paradigm^2^, refers to a performance cost associated with recently attended spatial locations^3^,^4^. In Posner and Cohen’s original experiments^1^,^5^, attention was first directed to one of two peripheral locations by an onset cue and then, a target appeared at the cued or the uncued location. Initially, response times (RTs) were shorter for targets at the cued location than those at the uncued location, an effect commonly attributed to the exogenous capture of attention by the cue^2^. However, when the cue-target onset asynchrony (CTOA) exceeded about 250 ms, RTs were longer for targets at the cued location than those at the uncued location. This inhibitory cueing effect was later termed as “inhibition of return” by Posner and colleagues^6^ to reflect their theoretical proposition that attention is inhibited from returning to previously attended locations.

As for the ecological function of IOR, Posner and Cohen suggested that IOR evolved to maximize the sampling of the visual environment^1^. This theoretical proposition is supported by subsequent observations of IOR in difficult visual search tasks that involve sequential shifts of attention^7^,^8^,^9^,^10^,^11^,^12^,^13^ (but see Wolfe and Pokorny^14^). While awake, humans normally make 2–3 rapid eye movements (saccades) every second^15^. Quick saccades direct the most sensitive part of the retina, i.e., the fovea, towards the visual space of interest, but it also constantly changes the retinal image of the environment. For IOR to aid visual sampling, it needs to be coded in spatiotopic or environment-centered coordinates, rather than retinotopic or eye-centered coordinates. Evidence for this prediction was first provided by Posner and Cohen^1^ and later confirmed by Maylor and Hockey^16^, with an improved methodology.

The task used by Maylor and Hockey and subsequent follow up studies^17^,^18^,^19^,^20^,^21^,^22^,^23^,^24^ is illustrated in Fig. 1a. The critical difference between this task and the classic cueing task is the introduction of a gaze-shift that intervened between the cue and the target. Following the presentation of the cue, subjects shift their gaze to a new location. Then, a target is presented at the cued spatial location (S-cued), a location that corresponds to the cued retinal locus (R-cued), or one of two mirror locations (S-mirror and R-mirror) that are used as baseline for calculating spatiotopic and retinotopic IOR effects. With this paradigm, Maylor and Hockey^16^ confirmed Posner and Cohen’s^1^ conclusion that IOR is coded in spatiotopic coordinates (see also Abrams and Pratt^17^). However, more recent studies have consistently shown that IOR is coded in both retinotopic and spatiotopic coordinates^18^,^20^,^21^,^22^,^23^,^24^.

IOR is not restricted to the cued location, but rather has a spatial gradient^16^,^25^,^26^,^27^,^28^. The diameter of the IOR gradient is estimated to be about 6–10° (visual angle)^16^,^25^,^28^ and has been shown to affect the processing of visual stimulus in the opposite visual field^25^. In the Maylor and Hockey task^16^, spatiotopic and retinotopic IOR effects are measured with the RT difference between cued and mirror locations (see Fig. 1b). As is clear from Fig. 1c, the R-mirror location is always more distant to the S-cued compared to R-cued location. Thus, a reliable RT difference between the R-cued and R-mirror locations can be observed even if there exists only a spatiotopic IOR gradient centered at the S-cued location. In the same vein, a reliable RT difference between the S-cued and S-mirror locations can be observed if there exists only a retinotopic IOR gradient centered at the R-cued location (see Fig. 1d). That is, the gradient of IOR makes it is difficult to tell if IOR effects reported in previous studies were coded in retinotopic or spatiotopic coordinates, or in both. One way to circumvent this serious methodological confound is to compare the RT of all four target locations to see which location has the longest RT. Indeed, the results of some studies show that RTs are longest at the cued spatial location^16^,^21^,^24^. This solution, however, is far from perfect because RTs to the target are likely affected by the intervening gaze-shift^18^,^21^. Furthermore, some studies presented onset stimuli at the second fixation to ease the intervening gaze-shift^21^. Such onset stimuli stimulate a retinal locus that is close to the R-cued and R-mirror locations and would affect the RTs at those locations if IOR can be coded in retinotopic coordinates.

The primary purpose of the present study was to clarify the nature of the IOR effects observed in the Maylor and Hockey task, by eliminating the above discussed methodological confounds. To achieve this goal, the Maylor and Hockey task was adopted, with the addition of no-cue trials as baseline for estimating IOR effects. In Mayor and Hockey’s original experimental task^16^, possible cue and target locations, and the direction of the intervening gaze shift were all fixed. Because it has been argued that a more dynamic task setting may facilitate the formation of spatiotopic representations^18^, the present study adopted a task that was more akin to that used by Mathôt and Theeuwes^21^. In this task, the position of the cue and the direction of the intervening gaze shift are randomly selected for each trial. The critical methodological difference between the present study and that of Mathôt and Theeuwes^21^ was that, although the positions of the cue and the target were calculated at the beginning of each experimental trial, the cue was not presented on half of the trials. The target that required saccadic responses was presented immediately following the intervening gaze-shift in Experiment 1, and was presented about 1.4 sec after the intervening gaze-shift in Experiment 2. For convenience, the time interval between the end of the gaze-shift and the onset of the target will be referred to as fixation-target onset asynchrony (FTOA). Previous work suggested different time courses for spatiotopic and retinotopic IORs^21^ and thus, it is necessary to examine the IOR gradient confound at both short FTOAs when IOR magnitude should be greater and long FTOAs when IOR magnitude should be less. The FTOAs varied from trial to trial. On average, the FTOA was 38 ms and 1424 ms for Experiments 1 and 2, respectively.

## Results

### Experiment 1: Short FTOA

#### Mirror locations as baseline

Analysis was first performed to reveal spatiotopic and retinotopic IOR effects, with the traditional method of estimating these effects, i.e., the RT differences between cued and mirror locations (see Table 1). Note that this analysis was performed only on trials with onset cues. A repeated measures ANOVA on the saccadic response times (SRTs) was performed, with factors coordinate system (spatiotopic vs. retinotopic) and cueing (cued vs. mirror). The results revealed a marginally significant main effect of coordinate system, *F*(1, 11) = 4.09, *p* = 0.068, 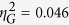. SRTs were generally shorter on retinotopic than on spatiotopic trials, largely consistent with that reported in previous work^18^,^21^. A significant main effect of cueing was observed, *F*(1, 11) = 19.42, *p* = 0.001, 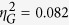, with longer SRTs observed for cued than for mirror locations, reflecting an overall IOR effect. Although the spatiotopic IOR effect (30 ms) was numerically larger than the retinotopic IOR effect (18 ms), the 2-way interaction failed to reach significance, *F*(1, 11) = 1.99, *p* = 0.186, 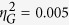.

#### No-cue trials as baseline

As is clear from the above analysis, SRTs were generally longer at spatiotopic locations (S-cued and S-mirror) because, relative to the intervening gaze-shift, saccades to those locations were “backward” saccades (see Wang and colleagues^29^, for a computational explanation). Thus, we cannot infer the IOR gradient directly from the raw SRTs. Note that, as is clear from Table 1, this SRT difference was also present on no-cue trials. By subtracting SRTs on no-cue trials from those on with-cue trials, a much more accurate estimate of IOR effect can be obtained. In addition, using no-cue trials as baseline also eliminates the concern that mirror locations may still lie on the IOR gradient and could lead to an underestimation of IOR effects. For distinguishing from the traditional method of estimating retinotopic and spatiotopic IOR effects, we will refer to the SRT difference between with-cue and no-cue trials as *pure IOR* (see Table 1). With no-cue trials, the mirror locations are no longer baselines for calculating IOR, but rather locations that can be used to reveal the gradient of spatiotopic and retinotopic IORs.

**Figure 1:**
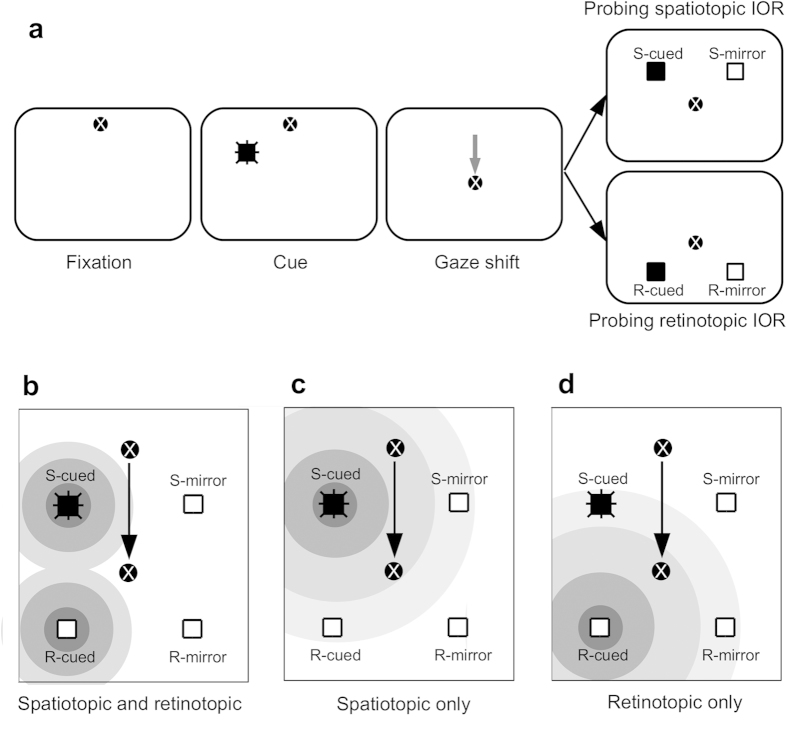
The cueing task used to reveal spatiotopic and retinotopic IORs. (**a**) An illustration of the task used in previous studies. Following an onset cue, the participants make an eye movement in response to the displacement of the fixation cross. This eye movement is necessary for dissociating the spatiotopic and retinotopic coordinates. After the eyes have arrived at the new fixation, a target that requires speeded response is presented at the cued spatial location (S-cued), a location that corresponds to the cued retinal locus (R-cued), or one of two mirror locations (S-mirror and R-mirror). (**b–d**) The IOR gradient makes the results of previous studies difficult to explain. While recent studies argued that IOR is coded in both spatiotopic and retinotopic coordinates (**b**), these results can also be observed if IOR is coded in spatiotopic (**c**) or retinotopic (**d**) coordinates only.

A repeated measures ANOVA on the SRTs was performed, with factors cueing (with- vs. no-cue) and target location (S-cued, S-mirror, R-cued, and R-mirror). The results revealed a significant main effect of cueing, *F*(1, 11) = 25.32, *p* = 0.000, 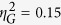, with longer SRTs observed for trials with cues, suggesting an overall IOR effect. The main effect of target location was significant, *F*(1, 11) = 7.85, *p* = 0.000, 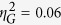. Interestingly, the 2-way interaction also reached significance, *F*(1, 11) = 6.52, *p* = 0.001, 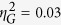, suggesting that IOR effects differed across all possible target locations. The IOR effects at the S-cue, R-cued, S-mirror and R-mirror locations were 54 ms, 44 ms, 25 ms and 17 ms, respectively. Planned contrasts suggest that all these IOR effects were statistically reliable, all *t’s* > 2.9, all *p’s* < 0.05, all Cohen’s *d*_*z*_ > 0.84, except for that at the R-mirror location, t = 1.81, *p* = 0.098, Cohen’s *d*_*z*_ = 0.52. As has been discussed, the gradient of IOR has confounded the theoretical explanation of all previous findings. If there exists only one IOR gradient centered at the S-cued location, as suggested in early works by Posner and Cohen^1^ and Maylor and Hockey^16^, IOR effects at the R-cued and S-mirror locations should be of equal strength because they were equally distant to the S-cued location (see Fig. 1c). This is, however, not the case. The IOR effect was much stronger for the R-cued than for the S-mirror location, *t*(11) = 4.44, *p* = 0.001, Cohen’s *d*_*z*_ = 1.28. This critical observation suggests that the IOR effect at the R-cued location was not simply the spillover of the spatiotopic IOR gradient centered at the S-cued location but also indicated a retinotopic contribution.

### Experiment 2: Long FTOA

The results of Experiment 1 were consistent with the theory that IOR is coded in both spatiotopic and retinotopic coordinates (see Fig. 1b). However, it is merely a speculation resulting from the comparison of the IOR effects at R-cued and S-mirror locations. Experiment 2 was carried out to dispel this doubt and to provide more direct evidence for the coexistence of spatiotopic and retinotopic IORs. The mean FTOA in Experiment 2 was 1424 ms. It is a well-documented fact that the magnitude of IOR decreases with time^5^. We speculated that, at a much longer FTOA, the gradient of spatiotopic and retinotopic IORs should contract following the decrease of center intensity and thus, would cease to influenced each other. If IOR is coded in both spatiotopic and retinotopic coordinates, statistically reliable IOR effects should be observed at both S-cued and R-cued locations, but not at mirror locations.

#### Mirror locations as baseline

A repeated measures ANOVA on the SRTs of with-cue trials was performed, with factors coordinate system (spatiotopic vs. retinotopic) and cueing (cued vs. mirror). The results revealed a significant main effect of cueing, *F*(1, 11) = 17.39, *p* = 0.002, 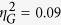, with longer SRTs observed for cued than for mirror locations, suggesting the observation of an overall IOR effect. The main effect of coordinate system did not reach significance, *F*(1, 11) = 0.17, *p* = 0.686, 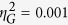, responses to the target were no longer slower for the S-cued and S-mirror locations. The 2-way interaction did not reach significance, *F*(1, 11) = 0.133, *p* = 0.723, 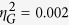, suggesting that the magnitude of retinotopic and spatiotopic IORs did not differ.

#### No-cue trials as baseline

As in Experiment 1, we also estimated the IOR effects with no-cue trials as baseline. A repeated measures ANOVA on the SRTs was performed, with factors cueing (with- vs. no-cue) and target location (S-cued, S-mirror, R-cued, and R-mirror). The results revealed a significant main effect of cueing, *F*(1, 11) = 25.32, *p* = 0.000, 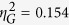, with longer SRTs observed for trials with cues, suggesting an overall IOR effect. The main effect of target location was significant, *F*(1, 11) = 7.85, *p* = 0.000, 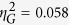. The 2-way interaction also reached significance, *F*(1, 11) = 6.52, *p* = 0.001, 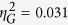. The IOR effects at the S-cue, R-cued, S-mirror and R-mirror locations were 11 ms, 16 ms, 6 ms and 6 ms, respectively (see Table 1). Planned contrasts revealed that IOR effects at the S-cued and R-cued locations were statistically reliable, all *t’s* > 3.0, all *p’s* < 0.05, Cohen’s *d*_*z*_ > 0.86, but those at the S-mirror location, *t*(*11*) = 1.64, *p* = 0.13, Cohen’s *d*_*z*_ = 0.47, and R-mirror location, *t*(*11*) = 1.55, *p* = 0.15, Cohen’s *d*_*z*_ = 0.45, were not different from zero. As in Experiment 1, the IOR effect was much stronger at the R-cued than at the S-mirror location, *t*(*11*) = 2.33, *p* = 0.040, Cohen’s *d*_*z*_ = 0.67.

It is important to note that, while reliable IOR effects were observed at the S-cued and R-cued locations, the IOR effects at mirror locations were no longer distinguishable from zero. Because the distance between S-cued and R-cued locations was the same as that between cued and mirror locations, the IOR gradients centered at the S-cued and R-cued locations did not overlap anymore. This pattern of results again suggests the existence of both spatiotopic and retinotopic IORs.

## Discussion

Using an experimental task originally devised by Maylor and Hockey^16^, recent studies consistently found that IOR can be coded in both spatiotopic and retinotopic coordinates. However, all of these studies have overlooked a methodological confound arising from the fact that IOR has a fairly large gradient that extends even to the opposite visual field^25^. The size of the spatiotopic and retinotopic IOR gradients is currently unknown and thus it is problematic to use control locations that are not distal “enough” from the cued location. The present study incorporated no-cue trials in the Maylor and Hockey task to address this issue and our results revealed both spatiotopic and retinotopic IOR effects.

### Saccades in reverse direction have longer latencies

Previous studies have frequently reported that saccades that reverse direction tend to have longer latencies than those that continue in the same direction^13^,^30^,^31^,^32^,^33^,^34^,^35^. Similar observations have been reported in studies that used the Maylor and Hocky task^18^,^21^ because, as illustrated in Fig. 1a, relative to the direction of the intervening saccade, saccades to the S-cued and S-mirror locations are roughly in the reverse direction (directional offset = 135*°*) whereas those to R-cued and R-mirror locations are roughly in the forward direction (directional offset = 45*°*). These behavioral observations have even been attributed to IOR^13^,^34^ or saccadic momentum^32^. Recent computational work^29^,^35^, however, suggests that this effect is likely the result of the residual activation in the superior colliculus (SC), associated with the immediate preceding saccade. Shortly after the eyes have landed at a new location, the SC activation responsible for the shift of gaze will not die out immediately, but rather will last for a while^36^. Because saccades made in natural conditions are typically small in size^37^,^38^, the residual activation is close to the fixation zone (the rostral pole of the SC) and will merge with fixation-related activity to cause an asymmetric activation of the SC. As a result, saccades that continue in the same direction as their preceding ones will have much shorter latencies. One critical prediction of this model-based explanation is that this effect is short-lived because the residual activation associated with the immediate preceding saccade decays quickly^29^,^35^. The results of the present experiments are consistent with this prediction. As clearly shown in Table 1, regardless of the presentation of the cue, SRTs were much shorter for saccades to R-cued and R-mirror locations, but only when the FTOA was short (Exp. 1). When the FTOA was relatively long (Exp. 2), no SRT difference was observed between S-cued, S-mirror and R-cued, R-mirror locations.

### Retinotopic IOR

As mentioned before, the observation of retinotopic IOR seems to contradict Posner and Cohen’s original functional explanation of IOR^1^. If IOR resides in retinotopic coordinates, objects that have not yet been attended in spatiotopic or environment-centered coordinates could also be inhibited while gaze shifts bring them to the inhibited retinal locus. Thus, retinotopic IOR may actually impede rather than facilitate visual foraging. This is one of the reasons we tried to determine if the IOR gradient has confounded previous observations of retinotopic IOR. As our results clearly confirmed its existence, the question of how IOR coded in retinotopic coordinates affects visual foraging remains. In the Maylor and Hockey task (see Fig. 1a), the intervening saccade is not made towards the cue. In normal search scenarios, however, a sudden onset usually captures not only attention but also the eyes, i.e., oculomotor capture^39^. In this case, the onset and the saccade towards it may evoke a) a retinotopic IOR that may discourage the eyes to repeat the vector of the preceding movement, and b) some residual activation in the SC that eases eye movements in the same direction (see the previous section for a discussion). It is likely that these two processes will cancel each other, leaving only the spatiotopic IOR at previous fixations to bias orientation.

### Spatiotopic IOR and predictive remapping

**Figure 2:**
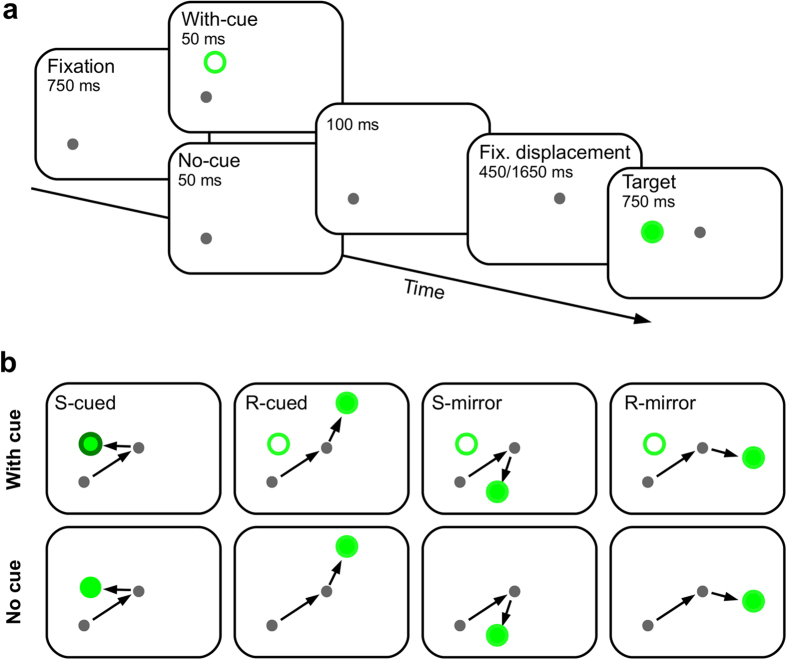
Task procedure and all possible trial types. (**a**) Task procedure. Note that the onset cue was presented on half of the trials, see text for details. (**b**) The presence of the onset cue and the position of the target relative to the cue yielded a total of 8 possible trial types. This figure is for illustration purpose only; the direction of the fixation displacement was randomly chosen for each trial.

One major challenge to the visual system is to integrate constantly changing retinal images into a stable representation of the visual space. One potential solution to this problem is predictive remapping^40^,^41^,^42^. The essence of the predictive remapping idea is that within retinotopic maps information is transferred in the opposite direction of an impending eye movement, so as to compensate for the upcoming retinal image displacement. Mathôt and Theeuwes^21^ recently showed that, immediately following the execution of an eye movement, IOR is coded in retinotopic coordinates only; at longer post-saccadic intervals, however, IOR is coded in spatiotopic coordinates only. These results seem to suggest a gradual transfer of IOR on retinotopic maps after rather than before gaze shifts. Although the methodology of the present experiments was very similar to that of Mathôt and Theeuwes^21^, Experiment 1 revealed that robust spatiotopic IOR effects can be observed immediately following gaze shifts, suggesting that the remapping of IOR may occur before rather than after gaze shifts^18^,^22^. To clarify if the observed spatiotopic IOR effect was the result of predictive remapping, future studies need to also probe the remapped retinal locus^42^ before the initiation of the intervening saccade. In the illustration of Fig. 2b, relative to the first fixation dot, the remapped retinal locus is on the same side as the cue, but is 135*°* away.

### Baseline matters

One important implication of the present experiments is that both spatiotopic and retinotopic IORs may have been underestimated in previous work. When the FTOA was 38 ms (Exp. 1), the spatiotopic and retinotopic IOR effects were 30 ms and 18 ms, respectively, with mirror locations as baseline. When no-cue trials were used as baseline, however, the spatiotopic and retinotopic IOR effects at the S-cued and R-cued locations increased to 55 ms and 43 ms, respectively. When the FTOA increased to 1424 ms (Exp. 2), this baseline difference disappeared, possibly because the IOR gradients shrunk and did not affect the mirror locations anymore.

One might argue that using no-cue trials as baseline may lead to an overestimation of IOR effects if spatiotopic and retinotopic IORs have additive behavioral effect. We believe this is less of a concern. In a recent study, Krüger and Hunt^20^ modified the Maylor and Hockey task to include trials on which the intervening saccade was not required. This design allowed them to directly compare retinotopic and spatiotopic IOR effects to the IOR effects measured in typical cueing tasks, in which the retinotopic and spatiotopic coordinates overlap. If retinotopic and spatiotopic IORs have additive behavioral effects, it is expected that a stronger IOR effect should be observed when the retinotopic and spatiotopic coordinates overlap. They found that, although the retinotopic IOR effect was numerically smaller than the spatiotopic IOR effect and the IOR effects in overlapping coordinates, no reliable difference was observed among these three effects.

## Methods

The experiments reported here were approved by the Institutional Review Board of Center for Cognition and Brain Disorders at Hangzhou Normal University and were carried out in accordance with the guidelines expressed in the Declaration of Helsinki. Written informed consent was obtained from all participants.

### Participants

Twenty-four students from Hangzhou Normal University participated this experiment in exchange for monetary compensation (40 Yuan/hour). Half of them were tested in the short FTOA experiment (Exp. 1; 7 females and 5 males; ages ranged from 20 to 26 years, mean age: 23.4 years) while the others were tested in the long FTOA experiment (Exp. 2; 8 females and 4 males; ages ranged from 19 to 25 years, mean age: 21.8 years). All participants reported normal or corrected-to-normal vision, and were naive with respect to the purposes of the present study.

### Apparatus

All visual stimuli were presented against a black background (1.95 cd/m^2^) on a 19-inch CRT monitor. The visible area of the monitor display measured 31.8° × 23.8° at a viewing distance of about 64 cm (maintained with the use of a chin rest). Stimulus presentation and data registration were controlled by a Mac Mini, running custom scripts written in Python. Eye movements were monitored and recorded with an Eyelink 1000 (SR Research®) eye-tracker. The spatial resolution of this eye-tracker was 0.2° or better and the participant’s gaze position was sampled at 500 Hz.

### Design and Procedure

The experimental task was similar to that of Mathôt and Theeuwes^20^. Drift-correction was performed at the beginning of each trial. A gray cross served as the drift-correction target and remained visible until the participant’s gaze was within 1° (visual angle) of it and he/she pressed the space bar simultaneously. The sequence of events in a single trial is illustrated in Fig. 2a. Each trial started with the presentation of a gray fixation dot (diameter = 0.5°, Weber contrast = 8.69). After 750 ms, an onset cue (an empty green circle, diameter = 1°, Weber contrast = 59.51) was presented for 50 ms, at a randomly chosen location on an imaginary circle (radius = 4°) centered at the fixation dot. After another 100 ms, the fixation dot was displaced for 6° in a direction that was 45° (polar angle) away from the cued location. Participants needed to move their eyes to follow this displacement with 450 ms. Failure to do so would lead to a trial being aborted and later recycled. In the short FTOA experiment (Exp. 1), the target (a green disk, diameter = 1°, Weber contrast = 59.51) was presented immediately after the eyes had arrived at the displaced fixation. In the long FTOA experiment (Exp. 2), however, the target was not presented until 1650 ms had elapsed since the displacement of fixation dot. The target was presented for 750 ms and the participants were instructed to saccade to it as quickly as possible. The target could appear at the cued location (S-cued), a location that was 90° (polar angle) away from but was on the same side as the cued location (R-cued), or at one of the locations mirroring the S-cued and R-cued locations. The R-cued location corresponds to the cued retinal locus following the gaze shift. The location of the saccade target was used as the starting location for the next trial.

The most important methodological difference between the present study and all previous studies that used the Maylor and Hockey task was that the cue was not presented on 50% of the trials. The experimental script calculated the location of the cue, the location of the displaced fixation, and all four possible target locations at the beginning of each trial. On no-cue trials, however, the cue was not presented. The relationship between the cue and the target stimulus, and the presence of the cue stimulus led to a total of eight different trial types (Fig. 2b). Each trial type was tested for 32 trials, adding up to a total of 256 trials. If the participant’s gaze deviated more than 1° from the first fixation dot during fixation, or landed at a location that was more than 2° away from the displaced fixation dot, auditory and visual feedbacks were given and the trial was aborted. All aborted trials were discarded and retested in a random order later, until all trials were completed successfully. Trials with and without cues were intermixed within blocks of trials.

### Data analysis

Saccadic response times (SRTs) were defined as the time interval between the onset of the target and the initiation of the saccade that followed. Saccade initiation was detected online, with a velocity threshold of 30°/s and an acceleration threshold of 8000°/s^2^.

Only successfully completed trials were considered in our analyses. A trial was excluded from analyses if a) the eyes missed the target for more than 2° (19.91% and 13.28% for Experiments 1 and 2, respectively), or b) the saccadic response time (SRT) was extremely short (<90 ms; 4.21% and 1.36%) or was 2.43 SDs above the mean of the same condition (1.68% and 1.66%)^43^. A visual signal takes about 70 ms to reach the SC^44^ and the efferent delay of stimulation delivered to the SC is about 20 ms^45^. Trials with SRTs shorter than 90 ms were first excluded because the recorded saccades were unlikely to be responses to the target. After data cleansing, 78.32% and 85.03% of the trials remained in Experiments 1 and 2, respectively.

All statistical analyses were conducted in R^46^ and the significance level was set at 0.05. Repeated measures ANOVAs and paired t-tests were performed on the SRTs to reveal spatiotopic and retinotopic IORs. For the ANOVAs, we report generalized eta squared 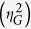
47 as an effect size measure because it provides comparability across between- and within-subjects designs^48^.

## Additional Information

**How to cite this article**: He, T. *et al.* Environment- and eye-centered inhibitory cueing effects are both observed after a methodological confound is eliminated. *Sci. Rep.* **5**, 16586; doi: 10.1038/srep16586 (2015).

## Figures and Tables

**Table 1:** Mean target SRTs (ms) of all conditions in Experiments 1 and 2.

FTOA		S-cued	S-mirror	Cued - mirror	R-cued	R-mirror	Cued - mirror
38 ms (Exp. 1)	With-cue	270 (39.7)	238 (35.3)	32 (17.7)^***^	245 (45.1)	224 (61.5)	21 (31.9)^*^
	No-cue	216 (36.5)	213 (36.9)	–	201 (43.1)	207 (39.9)	–
	*Pure IOR*	54 (32.7)^***^	25 (29.4)^*^	–	44 (29.2)^***^	17 (33.6)^*^	–
1424 ms (Exp. 2)	With-cue	201 (16.7)	193 (12.8)	8 (11.6)^**^	203 (15.7)	192 (16.6)	11 (15.2)^*^
	No-cue	190 (15.5)	187 (17.9)	–	187 (14.6)	186 (22.8)	–
	*Pure IOR*	11 (12.1)^*^	6 (12.7)	–	16 (13.3)^**^	6 (15.4)	–

IOR effects were estimated with the SRT difference between cued and mirror locations (as in previous work), or the SRT difference between with- and no-cue trials (pure IOR). Numbers in the parentheses are standard errors (SDs).

Note: ^*^p < 0.05, ^**^p < 0.01, ^***^p < 0.001.
